# Supplementary material for: Optical Properties of Red-Emitting Rb2Bi(PO4)(MoO4):Eu3+ Powders and Ceramics with High Quantum Efficiency for White LEDs
Source: Materials (Basel). 2019 Oct 8;12(19):3275. doi: 10.3390/ma12193275 (PMC6804156; doi:10.3390/ma12193275)
Supplement: Supplementary file 1 [file materials-12-03275-s001.pdf]

Supplementary Information

Optical Properties of Red-Emitting $Rb_2Bi(PO_4)(MoO_4):Eu^{3+}$ Powders and Ceramics with High Quantum Efficiency for White LEDs

Julija Grigorjevaite [1], Egle Ezerskyte [1], Agne Minderyte [1], Sandra Stanionyte [2], Remigijus Juskenas [2], Simas Sakirzanovas [1] and Arturas Katelnikovas [1,*]

[1] Institute of Chemistry, Vilnius University, Naugarduko 24, Vilnius LT-03225, Lithuania; julija.grigorjevaite@chf.vu.lt (J.G.); egle.ezerskyte@chf.stud.vu.lt (E.E.); agne.minderyte@chf.stud.vu.lt (A.M.); simas.sakirzanovas@chf.vu.lt (S.S.)

[2] Centre for Physical Sciences and Technology, Sauletekio Avenue 3, Vilnius LT-10257, Lithuania; sandra.stanionyte@ftmc.lt (S.S.) remigijus.juskenas@ftmc.lt (R.J.)

* Correspondence: arturas.katelnikovas@chf.vu.lt; Tel.: +370 697 23123

Received: 4 September 2019; Accepted: 4 October 2019; Published: date

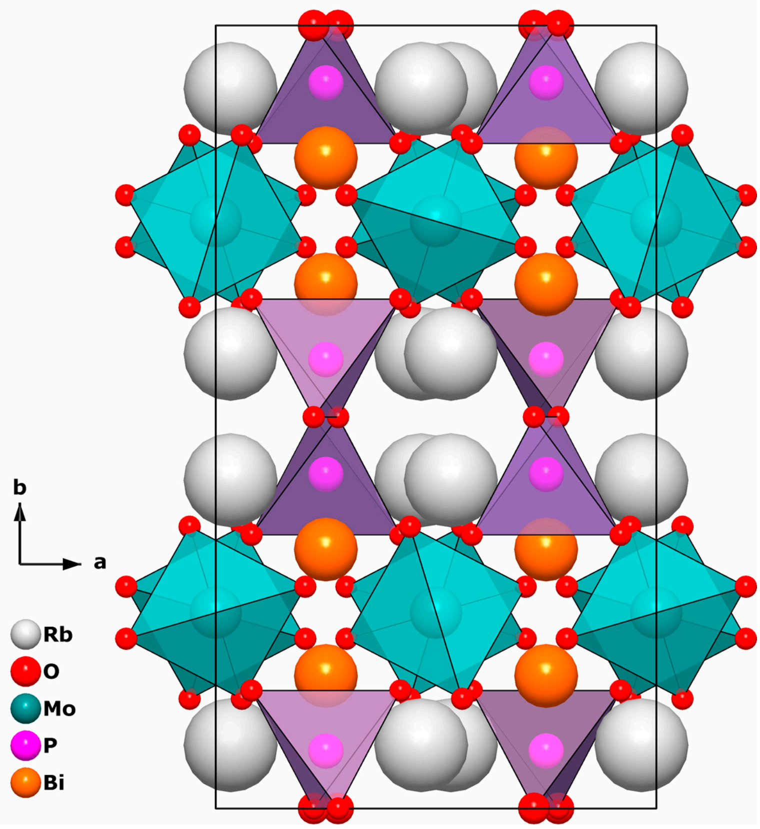

Figure S1. Unit cell of $Rb_2Bi(PO_4)(MoO_4)$ along the c-axis.

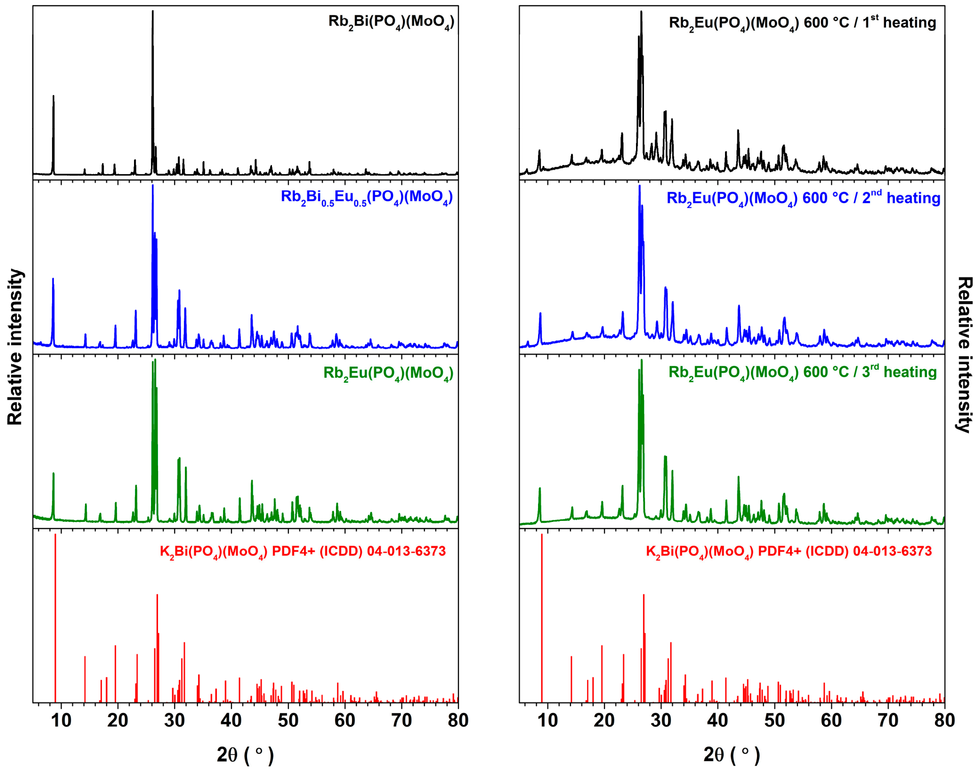

Figure S2. XRD patterns of $Rb_2Bi(PO_4)(MoO_4):Eu^{3+}$ as a function of Eu^{3+} concentration and heating time. The reference pattern of $K_2Bi(PO_4)(MoO_4)$ is given for comparison.

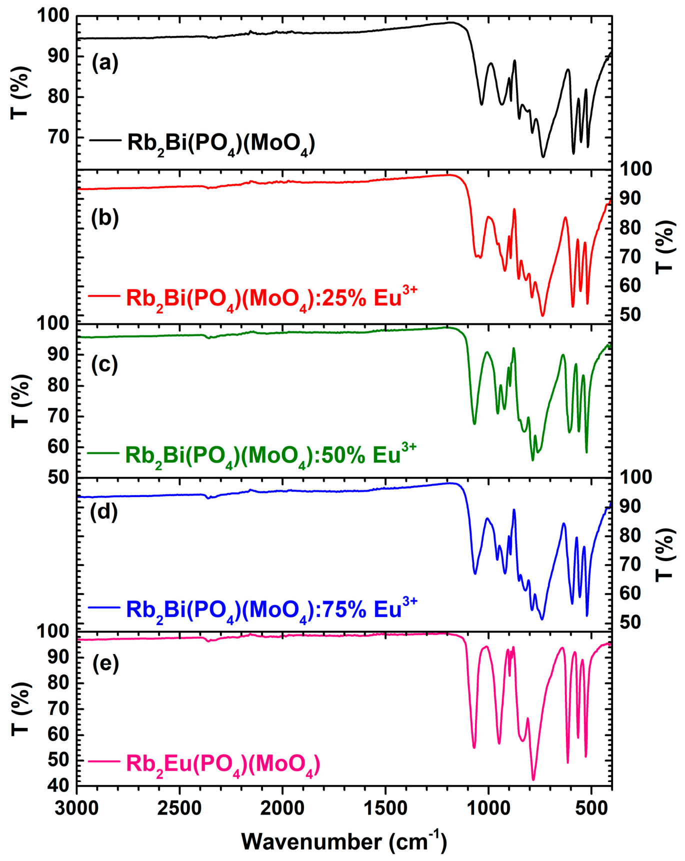

Figure S3. FTIR spectra of $Rb_2Bi(PO_4)(MoO_4):Eu^{3+}$ doped with 0% Eu^{3+} (**a**), 25% Eu^{3+} (**b**), 50% Eu^{3+} (**c**), 75% Eu^{3+} (**d**), and 100% Eu^{3+} (**e**).

Table S1. The weight of reagents used for the synthesis of $Rb_2Bi(PO_4)(MoO_4):Eu^{3+}$ powders.

Eu^{3+}, %	m(Rb_2CO_3), g	m(Bi_2O_3), g	m(Eu_2O_3), g	m(MoO_3), g	m($NH_4H_2PO_4$), g
0	0.5457	0.5505	-	0.3401	0.2718
1	0.5462	0.5455	0.0042	0.3404	0.2720
5	0.5482	0.5253	0.0209	0.3416	0.2730
10	0.5506	0.4999	0.0420	0.3432	0.2742
25	0.5582	0.4224	0.1063	0.3479	0.2780
50	0.5714	0.2882	0.2177	0.3561	0.2846
75	0.5851	0.1476	0.3343	0.3647	0.2914
100	0.5995	-	0.4568	0.3737	0.2986

Table S2. PL lifetime values of $Rb_2Bi(PO_4)(MoO_4):Eu^{3+}$ phosphors as a function of Eu^{3+} concentration and excitation wavelength.

Eu^{3+}	λ_{ex} = 265 nm		λ_{ex} = 393.5 nm		λ_{ex} = 464.5 nm	
(%)	τ (μs)	Std. dev. (μs)	τ (μs)		τ (μs)	Std. dev. (μs)
1	2424	2.4	1827	1.6	1814	1.7
5	2470	2.5	1846	1.6	1830	1.7
10	2420	2.4	1843	1.6	1843	1.6
25	2451	2.2	1893	1.6	1891	1.7
50	2300	2.1	2044	1.8	2057	1.8
75	2342	2.2	1955	1.7	1954	1.7
100	1973	1.8	1932	1.8	1947	1.7

Table S3. Temperature dependent PL lifetime values (λ_{ex} = 393.5 nm, λ_{em} = 615 nm) of $Rb_2Bi(PO_4)(MoO_4)$ phosphors doped with 1%, 50%, and 100% Eu^{3+}.

T	$Rb_2Bi(PO_4)(MoO_4)$:1% Eu^{3+}		$Rb_2Bi(PO_4)(MoO_4)$:50% Eu^{3+}		$Rb_2Eu(PO_4)(MoO_4)$	
(K)	τ (μs)	Std. dev. (μs)	τ (μs)	Std. dev. (μs)	τ (μs)	Std. dev. (μs)
77	1943	2.6	2131	2.4	1931	2.4
100	1937	2.5	2100	2.7	1860	2.4
150	1914	2.5	2059	2.6	1834	2.4
200	1891	2.5	2042	2.6	1864	2.4
250	1864	2.5	2030	2.6	1906	2.5
300	1840	2.4	2017	2.6	1942	2.5
350	1811	2.4	1995	2.6	1961	2.5
400	1786	2.4	1976	2.6	1965	2.5
450	1749	2.3	1953	2.5	1956	2.5
500	1712	2.3	1913	2.5	1905	2.5

Table S4. 1931 colour coordinates and LE values of synthesized phosphors as a function of Eu^{3+} concentration and excitation wavelength.

Eu^{3+} (%)	λ_{ex} = 265 nm			λ_{ex} = 393.5 nm			λ_{ex} = 465 nm		
	CIE 1931		LE	CIE 1931		LE	CIE 1931		LE
	x	y	(lm/W_{opt})	x	y	(lm/W_{opt})	x	y	(lm/W_{opt})
**1**	**0.64953**	**0.34998**	203	0.64840	0.35120	210	0.64767	0.35186	211
5	0.64933	0.35025	208	0.64663	0.35294	210	0.64727	0.35228	210
10	0.65127	0.34836	205	0.64841	0.35121	208	0.64896	0.35065	205

25	0.65170	0.34795	207	0.65025	0.34940	209	0.65055	0.34910	208
50	0.65130	0.34838	207	0.65099	0.34868	207	0.65209	0.34759	202
75	0.65168	0.34800	208	0.65100	0.34868	208	0.65155	0.34812	207
100	0.65083	0.34886	205	0.65152	0.34816	205	0.65252	0.34716	199

Table S5. CIE 1931 colour coordinates and LE values of synthesized phosphors as a function of Eu^{3+} concentration and temperature (λ_{ex} = 393.5 nm).

T (K)	$Rb_2Bi(PO_4)(MoO_4)$:1% Eu^{3+}			$Rb_2Bi(PO_4)(MoO_4)$:50% Eu^{3+}			$Rb_2Eu(PO_4)(MoO_4)$		
	CIE 1931		LE (lm/W_{opt})	CIE 1931		LE (lm/W_{opt})	CIE 1931		LE (lm/W_{opt})
	x	y		x	y		x	y	
**77**	**0.64998**	**0.34968**	192	0.64569	0.35362	198	0.65015	0.34951	194
100	0.65009	0.34958	192	0.64591	0.35341	199	0.65032	0.34935	194
150	0.65045	0.34923	193	0.64638	0.35298	200	0.65063	0.34904	196
200	0.65076	0.34891	194	0.64680	0.35259	201	0.65093	0.34875	196
250	0.65099	0.34868	194	0.64730	0.35213	202	0.65095	0.34873	197
300	0.65090	0.34877	195	0.64754	0.35193	203	0.65081	0.34886	198
350	0.65049	0.34917	196	0.64752	0.35196	204	0.65048	0.34918	199
400	0.64938	0.35026	199	0.64674	0.35273	206	0.64974	0.34990	201
450	0.64787	0.35172	199	0.64573	0.35370	208	0.64830	0.35129	203
500	0.64547	0.35406	200	0.64379	0.35557	212	0.64610	0.35344	207

Table S6. CIE 1931 colour coordinates and luminous efficacies (LE) of different thicknesses $Rb_2Eu(PO_4)(MoO_4)$ ceramics mounted on 375, 400, and 455 nm LEDs.

LED (nm)	Thickness (mm)	CIE 1931		LE (lm/W_{opt})
		x	y	
**375**	**0.36**	0.63214	0.34123	130
	0.53	0.64026	0.34323	182
	0.80	0.64588	0.34168	186
400	0.36	0.49987	0.24509	98
	0.53	0.55048	0.27854	135
	0.80	0.58266	0.29711	156
455	0.36	0.15295	0.04116	55
	0.53	0.16264	0.04726	63
	0.80	0.17756	0.05641	73
